# Molecular Mechanisms Regulating Organ-Specific Metastases in Epithelial Ovarian Carcinoma

**DOI:** 10.3390/cancers10110444

**Published:** 2018-11-15

**Authors:** Maria V. Barbolina

**Affiliations:** Department of Biopharmaceutical Sciences, University of Illinois at Chicago, Chicago, IL 60607, USA; mvb@uic.edu; Tel.: +1-312-355-0670

**Keywords:** ovarian cancer, organ-specific metastases, peritoneal adhesion, mesothelium, omentum, peritoneal wall, lymph node, liver, lung, gene expression

## Abstract

Epithelial ovarian carcinoma is the most predominant type of ovarian carcinoma, the deadliest gynecologic malignancy. It is typically diagnosed late when the cancer has already metastasized. Transcoelomic metastasis is the most predominant mechanism of dissemination from epithelial ovarian carcinoma, although both hematogenously and lymphogenously spread metastases also occur. In this review, we describe molecular mechanisms known to regulate organ-specific metastasis from epithelial ovarian carcinoma. We begin by discussing the sites colonized by metastatic ovarian carcinoma and rank them in the order of prevalence. Next, we review the mechanisms regulating the transcoelomic metastasis. Within this chapter, we specifically focus on the mechanisms that were demonstrated to regulate peritoneal adhesion—one of the first steps in the transcoelomic metastatic cascade. Furthermore, we describe mechanisms of the transcoelomic metastasis known to regulate colonization of specific sites within the peritoneal cavity, including the omentum. Mechanisms underlying hematogenous and lymphogenous metastatic spread are less comprehensively studied in ovarian cancer, and we summarize mechanisms that were identified to date. Lastly, we discuss the outcomes of the clinical trials that attempted to target some of the mechanisms described in this review.

## 1. Introduction

Ovarian carcinoma is the fifth leading cause of death from female cancers [1] and comprises several malignancies of epithelial and non-epithelial origins. Epithelial ovarian carcinoma (EOC) is the most predominant type, which, in turn, encompasses several distinct histotypes that are thought to originate in epithelial cells of the female reproductive tract [2,3,4]. High-grade serous ovarian carcinoma (HGSOC) is the most predominant histotype of EOC, and is thought to originate in epithelial cells of the ovaries and fallopian tubes.

HGSOC metastases spread via several distinct routes, including transcoelomic, hematogenous, and lymphogenous, with the former being the most predominant [5,6]. Transcoelomic spread refers to a route of tumor metastasis across a body cavity, such as the peritoneal cavity, as in the case of ovarian cancer. Transcoelomic metastases from ovarian cancer primarily seed the viscera of organs and tissues of the peritoneal cavity; metastasizing cells first attach to the mesothelial monolayer of intraperitoneal tissues and subsequently invade the extracellular matrix of the underlying stroma. The majority of patients with EOC are first diagnosed when peritoneal metastases have already formed, because the disease at early stages (when the tumor is confined to the ovary) is nearly asymptomatic.

These patients are typically treated by surgery and neoadjuvant chemotherapy (NACT) consisting of a combination of a platinum drug and a taxane. The Gynecologic Oncology Group defined optimal debulking as residual implants less than 1 cm [7]. Optimal debulking is often not possible due to the vast spread of metastatic lesions across the viscera of the peritoneal cavity [8]. Analysis of 3126 patients demonstrated that one-third each had complete resection, a small residual tumor burden of 1–10 mm, or macroscopic residual disease >1 cm in diameter [9], indicating that optimal resection could be achieved in approximately two-thirds of patients. In cases when optimal debulking is not feasible, the preferred treatment route includes interval debulking surgery after NACT [10,11]. Although most cases are initially responsive to chemotherapy, most become less and less sensitive with every recurrence, and eventually develop chemoresistance [12,13]. Moreover, metastatic ovarian cancer is thought to contain a subpopulation of cells with self-renewing capacity, or cancer stem cells, which are not affected by cytotoxic chemotherapy [14,15]. Patients who relapse more than six months after completion of platinum/taxane initial therapy are considered platinum-sensitive. Patients who respond to primary treatment and relapse within six months are characterized as platinum-resistant. Patients who relapse within 3 months of treatment are regarded as platinum-refractory [16]. The five-year survival for patients with advanced EOC is below 30%. Recurrent chemotherapy-resistant EOC is incurable. Moreover, peritoneal metastases are known to cause malnutrition and cachexia, which is associated with metabolic changes and bowel obstruction in patients with ovarian carcinoma [17,18,19]. Cachexia is strongly associated with worse prognosis [20,21,22].

For these reasons, the mechanisms regulating peritoneal metastasis from EOC are studied most extensively with the aim of identifying ways of preventing re-colonization of mesothelial linings and blocking or retarding the growth of intraperitoneal lesions using novel targeted molecular therapies that could be applied either alone or in conjunction with conventional chemotherapies. The hematogenous route of metastatic colonization also contributes to formation of intraperitoneal metastases [23,24]. The lymphatic system is often involved by the disseminating EOC cells as well [25,26,27,28]. Formation of thoracic metastases from EOC is thought to occur, in part, via direct extension of the peritoneal metastases or via lymphatics, although the relative contribution of these mechanisms is yet to be experimentally established.

In this review, we focus on the molecular mechanisms currently known to underlie the formation and development of organ-specific metastases from EOC.

## 2. Sites of EOC Metastases

Several studies assessed the patterns of metastatic spread from EOC at relapse, as well as at autopsy. These studies demonstrated that, although EOC typically colonized a wide variety of organs and tissues, there was not one single metastatic site that was always involved by metastasis in all studied cases. The main site of metastasis was the peritoneum, including the parietal and visceral peritoneum and omentum, which was involved in 77% of cases on average among several reports (ranging between 53% and 99%, Table 1) [25,26,27,29,30,31]. Other commonly colonized sites identified by autopsy studies included lymph nodes (38–77% of cases), large and small intestine (44–86% of cases), liver parenchyma (45–59% of cases), and lung (33–39% of cases). Pancreas, spleen, stomach, and ureter were involved in 3–24% of cases, while organs, such as the thyroid, bone, brain, skin, heart, breast, and kidney were typically colonized in 1–12% of the cases [25,26,27,32]. Notably, although distant metastases are rarely the main cause of death from metastatic EOC, their presence usually indicates worse prognosis [33,34]. For example, the presence of parenchymal splenic metastasis was an independent predictor of decreased overall survival [35]. Brain metastasis also correlated with worse prognosis among older patients [36,37].

Interestingly, the mutational status of tumor protein P53 (TP53) is linked with the propensity to seed either mainly peritoneal or peritoneal and distant metastases. Ninety-six percent of all cases belonging to HGSOC carry mutations in the *tp53* gene, which could occur at multiple locations within the gene sequence [34,38,39]. Null mutations of p53 were predictive of distant metastasis to liver, thorax, spleen, brain, and lymph nodes at initial diagnosis, and they occurred eightfold more frequently compared with cases containing either missense mutations of *tp53* or those expressing wild-type TP53 [34], although the detailed mechanisms are not known. Furthermore, cases with serous histology displayed slightly higher preponderance to having distant metastases (22 out of 66) in comparison to cases with other histologies, in which seven out of 35 cases had distant metastases [34].

In summary, the studies showed that metastases from ovarian carcinoma predominantly formed locally within the peritoneal cavity (peritoneum, omentum, and mesothelium); however, a large number of cases also presented with distant metastases, most commonly at lymph nodes, liver, and lung.

## 3. Mechanisms Regulating Transcoelomic Metastasis from EOC

**Figure 1:**
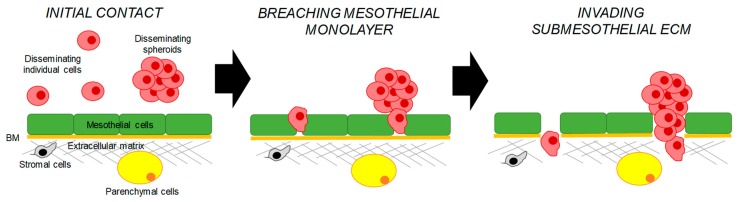
A scheme of the peritoneal metastasis through the transcoelomic route. Disseminating epithelial ovarian carcinoma (EOC) cells and spheroids are shown in mauve, mesothelial cells are shown in green, the basement membrane is shown in orange, stromal cells are shown in grey, parenchymal cells are shown in yellow, and the extracellular matrix is shown in grey.

Transcoelomic dissemination is thought to be the major route via which EOC metastasis spreads [6]. Peritoneal metastases can reach very large sizes and are often accompanied by the presence of the malignant ascites. These metastases are seeded by individual cells and multicellular aggregates or spheroids, which first adhere to mesothelial cells outlining the peritoneal cavity and then invade the submesothelial extracellular matrix (Figure 1). Studies of cell cultures of ovarian cancer cell lines demonstrated that cells could spontaneously detach from monolayers and remain as individual cells or form spheroids [40,41]. Recent in vivo studies demonstrated that spheroids predominantly formed by collective detachment from the primary tumor [40]. Mechanistically, it was suggested that a membrane type-1 matrix metalloproteinase plays a pivotal role in the spontaneous release of cell–cell adherent sheets, which later form spheroids [42]. Another study showed that individual cells could also aggregate together prior to mesothelial adhesion [43].

Progression of ovarian carcinoma is often accompanied by the presence of the malignant ascites, a fluid that accumulates within the peritoneal cavity [44,45]. It was estimated that more than one-third of ovarian cancer patients present with ascites at diagnosis, and nearly all contain ascites at the time of recurrence [46]. It is thought that ascites forms as a result of impaired drainage of the peritoneal cavity due to the obstruction of the lymphatic system by metastasizing tumor cells or an increased filtration rate to the peritoneal cavity [44]. The presence of the malignant ascites predicts poor prognosis in different ovarian cancer patient cohorts irrespective of the tumor’s histological type [47,48,49,50]. Ascites contains many soluble molecules, such as survival factors, including cytokines, chemokines, growth factors, and fragments of the extracellular matrix (ECM). In addition, ascites is an abundant source of the cells of the immune system, stromal and mesothelial cells, and cancer stem cells [46]. It is thought that the microenvironment within the ascites contributes to survival of the metastasizing ovarian cancer cells, provides support for tumor growth, and contributes to tumor heterogeneity [51].

### 3.1. Mechanisms Regulating Peritoneal Adhesion

The peritoneal mesothelium is a monolayer of mesothelial cells that lines the abdominal cavity [52]. Disseminating individual cells and spheroids adhere to the mesothelial lining of the intraperitoneal cavity to establish metastatic lesions. Studies described below demonstrated that both ovarian cancer and mesothelial cells play active roles in this process.

Mesothelial cells produce hyaluronan, which is thought to serve as a protective layer preventing attachment of the malignant EOC cells [53]. Inflammation associated with cancer may result in production of low-molecular-weight hyaluronan fragments and destruction of the protective hyaluronan coat consisting of the high-molecular-weight hyaluronan [54]. Different molecular pathways associated with both mesothelial and disseminating tumor cells could participate in promoting peritoneal adhesion.

The majority of EOC cases affect the elderly, as the median age at diagnosis is 63 [55]. Thus, several studies focused on characterization of senescent stromal cells, including peritoneal mesothelial cells in the metastatic process. Senescent mesothelial cells play a critical role in the development of peritoneal carcinomatosis in several cancer models, including ovarian cancer [56]. In studies of syngeneic ovarian cancer models, aged mice were more prone to formation of metastases than their younger counterparts [57]. Increased metastatic burden in aged mice also corresponded to significant changes in the cellular composition of the native immune system within the peritoneal adipose tissue such that the presence of tumor-infiltrating lymphocytes was higher and B-cell-related pathways were altered in comparison to younger mice [57].

EOC cells can co-opt mesothelial cells in order to facilitate peritoneal adhesion. It was demonstrated that EOC cells can secrete exosomes enriched for CD44 molecule (CD44). These exosomes could be internalized by mesothelial cells, resulting in the secretion of matrix metallopeptidase 9 (MMP-9), which helps EOC with cell invasion [58]. Mesothelial cells also secrete lysophosphatidic acid (LPA), which aids in mesothelial adhesion of EOC cells expressing receptors for LPA [59].

**Figure 2:**
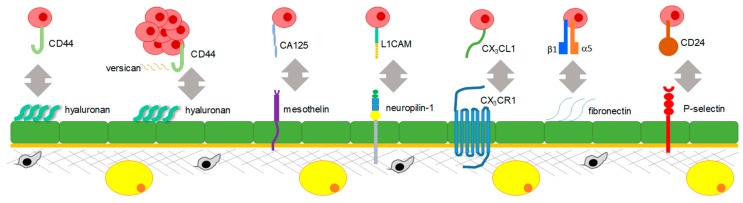
Molecular mechanisms regulating peritoneal adhesion. Disseminating epithelial ovarian cancer cells and spheroids are shown in mauve, mesothelial cells are shown in green, the basement membrane is shown in orange, stromal cells are shown in grey, parenchymal cells are shown in yellow, and the extracellular matrix is shown in grey. Only one interaction between a cancer cell and a mesothelial cell is shown for simplicity and a clearer presentation of the known mechanisms. CD44: CD44 molecule; CA125: mucin 16, cell surface associated or ovarian carcinoma antigen CA125; L1CAM: L1 cell adhesion molecule; CX_3_CL1: C-X3-C motif chemokine ligand 1; CX_3_CR1: C-X3-C motif chemokine receptor 1; CD24: CD24 molecule.

Numerous in vitro and in vivo studies used different experimental approaches to show that disseminating ovarian cancer cells themselves express a number of membranous receptors and adhesion molecules that facilitate their adhesion to mesothelial cells expressing ligands for these receptors (Figure 2).

The first interaction reported to facilitate peritoneal adhesion was between hyaluronan (HA) expressed by mesothelial cells and CD44 expressed by EOC cells [60,61]. It was also reported that extracellular tissue transglutaminase (TG2) expressed by ovarian cancer cells results in upregulation of CD44, which further promotes peritoneal adhesion [62]. Reduction of expression of a secreted glycoprotein, versican, which is thought to facilitate the CD44–HA interaction, in EOC cells, resulted in reduction of tumor formation by individual cells and abrogation of metastatic ability of spheroids [63,64]. These studies revealed an important role of CD44 and molecular interactions supporting its function, notably, TG2 and versican, in mesothelial adhesion. Further studies indicated that treatment with neutralizing anti-CD44 antibodies reduced tumor burden on the peritoneal wall and diaphragm in a xenograft mouse model [65], suggesting that targeting CD44 is a promising approach for the reduction of peritoneal tumor.

Other molecular interactions supporting peritoneal adhesion are fostered by a glycoprotein on the surface of EOC cells, ovarian carcinoma antigen CA125 (MUC16), and mesothelin expressed on the surface of mesothelial cells, both of which were demonstrated by in vitro studies using EOC cell lines [66,67].

A series of in vitro studies that utilized EOC cell lines and mesothelial cells demonstrated that neuropilin-1 expressed in mesothelial cells can interact with a glycoprotein expressed in EOC cells, the L1 adhesion molecule (L1CAM) [68].

Both in vitro studies of EOC and mesothelial cell co-cultures and in vivo studies of short-term adhesion and survival xenograft studies showed that a chemokine receptor fractalkine (CX_3_CR1) expressed in EOC cells can interact with its ligand CX_3_CL1 expressed (in its transmembrane form) by peritoneal mesothelial cells [69,70].

Studies using co-cultures of EOC cells and mesothelial cells pre-treated with β1-integrin-specific neutralizing antibodies demonstrated that β1-integrins expressed by EOC cells could interact with fibronectin expressed by mesothelial cells [71]. The role of α5β1-integrin-mediated adhesion of EOC cells to fibronectin-expressing mesothelial cells was further confirmed with a series of in vitro experiments utilizing primary mesothelial cells as well as in vivo using xenograft models of the disease [72]. The latter study highlighted the role of EOC cells in inducing fibronectin expression in mesothelial cells via transforming growth factor beta 1 (TGFβ1)-mediated signaling. 

Another interactive loop facilitating peritoneal adhesion is initiated by alternatively activated macrophages (AAMs) occurring in the peritoneal microenvironment of EOC, which involved stimulation of expression of a calcium-dependent receptor, P-selectin, on mesothelial cells by a C–C motif chemokine ligand 4 (CCL4 or MIP-1β) secreted by the AAMs; EOC cells expressing CD24 interacted with P-selectin-expressing mesothelial cells, as demonstrated by ex vivo and in vivo studies of a syngeneic ovarian cancer model [73].

Once metastasizing EOC cells adhered to the mesothelial monolayer, to anchor metastatic lesions, they need to disrupt the mesothelial lining and invade submesothelial parenchymal tissues consisting of the ECM, stromal cells, and cells of the organ parenchyma. EOC cells express various molecules that assist their invasion into the parenchyma of the organs and tissues to which they have adhered. Overexpression of alcohol dehydrogenase 1B (ADH1B) was demonstrated to regulate EOC cell attachment and clearance of the mesothelial lining, as well as subsequent matrix invasion [74]. Adherent spheroids could utilize integrin- and talin-dependent activation of myosin and traction force to clear the mesothelial monolayer [75]. Expression of a transmembrane glycoprotein prominin-1 (PROM1) correlated with the ability of EOC cells to adhere to and clear the mesothelial monolayer as well [76]. It was shown that N-cadherin, but not E-cadherin, is essential for the lateral dispersal of spheroids onto extracellular matrix and invasion; individual cells also depended on N-cadherin for their dispersal and penetration into the collagen gels [77].

After breaching the mesothelial monolayer, ovarian cancer cells quickly adhere to the submesothelial matrix, which is predominantly composed of collagens type I and III, using both α2β1- and α3β1-integrins [78,79]. MT1-MMP is a major interstitial collagenase enabling invasion and anchorage of metastatic ovarian cancer cells in the submesothelial matrix [80].Three-dimensional collagen I is instrumental in upregulating the transmembrane collagenase membrane type 1 matrix metalloproteinase (MT1-MMP) via several mechanisms, including integrin-dependent activation of an Src proto-oncogene, non-receptor tyrosine kinase (Src)-dependent pathway, and subsequent induction of a transcription factor early growth response 1 (EGR1), as well as matrix rigidity-dependent activation of wingless (Wnt) signaling through downregulation of dickkopf-1 expression [81,82]. Epidermal growth factor receptor (EGFR)-dependent modulation of MT1-MMP surface dynamics was also found to contribute to transition to a more invasive phenotype of ovarian cancer cells [83]. 

In summary, several molecular interactions between cancer and mesothelial cells establish successful cell–cell adhesion during mesothelial adhesion. Disseminating cancer cells take advantage of secreted molecules produced by mesothelial cells and can reprogram their gene expression to aid peritoneal adhesion. Likewise, aging can amplify the process of peritoneal carcinomatosis by providing more permissive conditions for cancer cell adhesion. Importantly, EOC cells themselves express proteins that enable their attachment and tissue invasion.

### 3.2. Mechanisms Regulating the Transcoelomic Omental Metastasis

The omentum is a peritoneal fold that connects the stomach with abdominal organs [84]. The omentum functions to protect and support abdominal organs and to limit intraperitoneal infection. In addition to a mesothelial monolayer covering this tissue, omentum mainly consists of adipocytes. Other prominent structures within the omentum are milky spots that are the areas of lymphoid tissue containing macrophages, lymphocytes, and mast cells [85]. The omentum also contains other stromal cells, such as fibroblasts, and it is supplied by the gastroepiploic arteries [86]. Studies showed that invading EOC cells successfully establish metastatic lesions in the omentum by taking advantage of the unique microenvironment within this tissue [87,88].

In metastasis from EOC, omentum plays a central role as one of the major tissues hosting peritoneal metastatic lesions [5,25,27,32]. Omentum is also a major site of recurrent metastasis in patients whose omentum was not completely resected. According to the current standard of care, omentum may be partially or completely resected in medically fit patients in the process of the debulking surgery depending on the degree of its involvement with the metastasis [89]. Due to the importance of omentum as a major secondary site, several studies addressed the mechanisms supporting survival and proliferation of metastatic EOC cells within the omental tissues, and uncovered the role of various omental stromal cells in supporting this process.

Recent studies suggested that, as cells detach from the primary tumor and become suspended in the ascites, they undergo a metabolic shift from glycolysis to lipid metabolism, which later affords and facilitates their survival within the omental tissue [90]. Metastasizing EOC cells are also attracted to the omentum by adipokines expressed by the adipocytes, such as adiponectin, interleukin-6 (IL-6), interleukin-8 (IL-8), C–X–C motif chemokine ligand 1 (CXCL1, GRO-α), and others [87,91].

Milky spots are mainly composed of macrophages and lymphocytes, and represent initial lymphatics of the omentum that drain into lymph collectors [92]. Preclinical studies that used ex vivo and in vivo syngeneic and xenograft mouse models demonstrated that disseminating EOC cells can lodge onto milky spots and further spread through the adipose-rich tissue [88,93]. In vivo studies with both syngeneic (ID8 mouse-derived ovarian cancer cell line in C57BL/6 mice) and xenograft (Caov-3, HEYA8, and SKOV3i.p.1 human-derived ovarian cancer cell lines in athymic nude mice) models of ovarian carcinoma suggested that disseminating cells preferentially lodge onto milky spot-containing adipose tissue as opposed to peritoneal fat, while the number and size of the milky spots did not depend on the mouse genetic background [93]. The study also showed that conditioned media collected from milky spot-containing adipose tissue significantly increased cell migration in comparison to the conditioned media from milky spot-deficient adipose tissue [93].

Once EOC cells lodge onto the omentum, proximity to adipocytes results in upregulation of fatty-acid-binding protein 4 (FABP4) and a fatty-acid receptor CD36, followed by transfer of lipids from adipocytes to EOC cells, and induction of lipolysis in adipocytes and β-oxidation in cancer cells [87,94]. Interaction of EOC cells with mesothelial cells reduced expression of microRNA-193 (miR-193) in the former, resulting in increased ability to colonize the omentum [95]. Consistent with cancer cell utilization of lipids stored in adipocytes as an energy source, a study that described the role of milky spots in metastatic colonization of the omentum also reported reduction of the adipose tissue as the tumors grew over time [93].

Fibroblasts in omentum also play a prominent role in regulating this organ-specific metastasis. A study uncovered interaction between tumor necrosis factor alpha (TNFα), transforming growth factor alpha (TGFα), and epidermal growth factor receptor (EGFR); this TNFα–TGFα–EGFR interacting loop is thought to form between EOC cells and fibroblasts that reside in omentum, and it is suggested that it functions to promote peritoneal metastasis [96].

Interactions between chemokine receptors expressed by cancer cells and their corresponding chemokines at the metastatic sites was suggested to regulate homing of metastasizing cells to their niches. Among these interactions, association between the C–X–C motif chemokine receptor 4 (CXCR4) and its ligand, stromal-derived factor 1, was demonstrated to regulate pro-metastatic functions of cells from several cancer types, including ovarian [97,98,99,100]. A specific inhibitor of CXCR4, AMD3100, nearly completely blocked EOC cell dissemination to the omentum in a rodent syngeneic model, supporting the importance of this chemokine axis in development of the omental metastasis [101]. Another study demonstrated that omentum-secreted IL-8 and GRO-α can activate C-X-C motif chemokine receptor 2 (CXCR2) in ovarian carcinoma cells and facilitate EOC cell spreading in the peritoneal cavity [91].

Thus, studies of the mechanisms of omental metastasis to date demonstrated important roles of the omentum itself and metastasizing EOC cells in facilitating formation and development of this major type of metastatic lesions.

### 3.3. Mechanisms Regulating Transcoelomic Metastasis to Other Intraperitoneal Organs and Tissues

The mechanisms regulating organ-specific intraperitoneal dissemination to other organs, including peritoneal wall, viscera of the bowels, viscera of the liver, etc., are less well understood as compared to the mechanisms regulating formation of the omental metastasis. Several studies uncovered the pivotal role of the chemokine–receptor interactions in regulating these organ-specific metastases. Inhibition of CXCR4 with AMD3100 significantly reduced colonization of the colon, peritoneal wall, diaphragm, and liver [101]. Downregulation of another chemokine receptor, X-C motif chemokine receptor 1, or lymphotactin (XCR1), almost completely abrogated colonization of diaphragm and peritoneal wall [102]. Further, it was demonstrated that yet another chemokine axis, between fractalkine (CX_3_CL1) and its receptor (CX_3_CR1), regulates dissemination of the CX_3_CR1-positive EOC cells to the surfaces of the CX_3_CL1-positive tissues, including peritoneal wall, diaphragm, liver, mesentery, and retroperitoneal kidneys [69,70].

## 4. Mechanisms Regulating Hematogenous Metastasis from EOC

Peritoneovenous shunting is a procedure in which a shunt could be used to return the peritoneal fluid from the peritoneal cavity into veins, such as the superior vena cava or the internal jugular vein, by means of a one-way valved anastomosis [103,104,105]. This method was attempted on a cohort of patients with ovarian cancer and other malignancies who had intractable ascites for the purpose of palliative care [106,107]. A study that described the autopsy findings of the patients that underwent this procedure concluded that most patients either did not develop distant metastases or grew very small isolated lesions as a result of this procedure [108]. In the ovarian cancer field, this was interpreted as suggesting that the hematogenous route has little relevance as a mechanism via which the metastasis forms. However, a close examination of the presented data suggests that this conclusion was overgeneralized. Eight out of nine ovarian cancer patients did not survive longer than about four months on average (survival ranged from one to seven months) after the initiation of this procedure; moreover, even over this short period of survival, evidence of distant metastases at lung, liver, spleen, brain, and other distant sites was found in three of the eight patients. Only one out of nine ovarian cancer patients survived for 27 months after the procedure without developing distant metastasis [108,109]. Importantly, although distant metastases were not the cause of death in this study, they did occur, even though all but one patient survived between one and seven months after insertion of the shunts.

In another patient-based study that focused on investigating the outcomes of inferior vena cava filter placement in patients with epithelial ovarian, fallopian, and primary peritoneal cancer, the authors reported that patients who underwent this procedure had significantly lower survival and significantly higher incidence of deep vein thrombosis and distant metastasis [110], supporting the role of a hematogenous route in seeding distant metastases from EOC. Additionally, seeding of distant organs, including brain [36], is likely to occur via this mechanism.

Experimentally, evidence of development of the hematogenous metastasis within the omentum was recently presented [23]. A novel parabiosis mouse model was used to demonstrate that the molecular interaction between the Erb-B2 receptor tyrosine kinase 3 (ERBB3) expressed by ovarian cancer cells and its ligand neuregulin-1 expressed by the omentum is the main driving force of the hematogenously spread omental metastasis. Parabiosis is a surgical union of two organisms that allows sharing of the blood circulation [111]. In the study on ovarian carcinoma, the parabiosis model was created by excising the skin of female mice from the shoulder to the hip joint followed by surgical anastomosis to make new connections between blood vessels of pairs of mice [23].

In another study, three approaches were employed to investigate the role of the hematogenous route of EOC metastases, including an intravascular tail-vein injection of ovarian cancer cells, as well as subcutaneous engraftment of murine and human tumors. Primary ovarian cancer cells were co-injected with mesenchymal stem cells subcutaneously. To promote formation of blood vessels in the tumor, human infantile hemangioma stem cells were co-injected as well. This protocol resulted in 100% engraftment rate and macroscopic ovarian metastases by the time of sacrifice [24]. The authors observed development of tumors not only within the ovary, but also at other distant sites, including the lung [24], further supporting existence of mechanisms driving hematogenous dissemination to different organ sites. A study on the role of CXCR4 in EOC metastases showed that downregulation of CXCR4 by short hairpin RNA (shRNA) resulted in a robust reduction of the circulating tumor cells, suggesting a possible role of the stromal cell-derived factor 1 (SDF1)/CXCR4 axis in the hematogenous route of dissemination [112]. 

In summary, many patient studies reported occurrence of the distant metastasis, which could have arrived at these sites, notably the brain, likely via the hematogenous route. Although these distant hematogenously spread metastases are not considered to be the cause of death from EOC by themselves, their presence is significantly correlated with worse survival. Presently, the peritoneal metastases from EOC is still an unsolved problem in clinical management of this disease. However, it is very likely that continuous progress in the treatment of the peritoneal metastasis and increased survival could allow for more time for development of the distant metastasis, which could become clinically relevant in long-term survivors of metastatic EOC.

## 5. Mechanisms Regulating Lymphatic Metastasis from EOC

The International Federation of Gynecology and Obstetrics (FIGO) ovarian cancer staging states that metastatic involvement of the retroperitoneal lymph nodes indicates FIGO Stage IIIC of the disease, and colonization of the inguinal lymph nodes and lymph nodes outside of the abdominal cavity by metastases signifies Stage IVB of the disease [113]. Although EOC metastases frequently involve lymph nodes, autopsy studies reported that the frequency of colonization differed by their anatomic location with the abdominal lymph nodes being most frequently colonized among others (Table 2).

Vascular endothelial growth factor receptor 3 (VEGFR3) is the major receptor involved in lymphangiogenesis and maintenance of the lymphatic endothelium [114]. The ligands activating this receptor are vascular endothelial growth factors C and D (VEGFC, VEGFD). Immunohistochemical analysis of expression of VEGFA, VEGFC and VEGFD in ovarian carcinoma patients, most of which (92/100) were diagnosed with FIGO Stage III disease with retroperitoneal metastases or those with predominantly intraperitoneal metastasis, demonstrated that high expression of VEGFC corresponded to the presence of the retroperitoneal metastasis, while low VEGFC correlated with mostly intraperitoneal metastatic spread, supporting the role of VEGFC–VEGFR3 interaction in EOC cell tropism to the lymph node. High VEGFC also correlated with shorter overall survival [115]. Another study was performed to characterize the patterns of expression of the ubiquitin-specific protease 7 (USP7); it was found that high expression of USP7 significantly correlated with lymph node metastases [116]. Upregulation of focal adhesion kinase (FAK) in EOC cells strongly correlated with incidence of lymph node metastases as well [117].

In summary, lymphatic involvement is correlated with worse outcomes. Patient studies demonstrated preferential colonization of the abdominal over other lymph nodes in the human body.

## 6. Targeted Therapies in Ovarian Cancer

Development of targeted therapies against ovarian carcinoma, although still mainly at the stage of characterization of new potential targets, is an actively growing field. Several proteins that were demonstrated to play a role in progression and metastasis of ovarian cancer were or currently are investigated as novel drug targets in clinical trials. The targeting agents used in these studies vary widely from small-molecule inhibitors to monoclonal antibodies, antibody–drug conjugates, and immunotherapy.

For example, a CD44-targeting compound SPL-108 is being investigated in conjunction with paclitaxel in a phase I trial against epithelial ovarian carcinoma (ClinicalTrials.gov identifier: NCT03078400).

Several clinical trials are attempting to target mesothelin. Among those, one clinical trial (ClinicalTrials.gov identifier: NCT03692637) that is currently in phase I is aiming to use anti-mesothelin chimeric antigen receptor (CAR) natural killer (NK) cells in epithelial ovarian carcinoma. This study is taking advantage of the new targeting technology based on the use of CAR-NK therapy consisting of chimeric antigen receptor (CAR)-expressing natural killer cells of the immune system [118]. Another mesothelin-targeting approach is being investigated in a phase I clinical trial involving patients with recurrent mesothelin-expressing platinum-resistant ovarian cancer, and this study will test the efficacy of anetumab ravtansine in combination with polyethylene glycol (PEG) PEGylated liposomal doxorubicin (ClinicalTrials.gov identifier: NCT02751918). Anetumab ravtansine is an antibody–drug conjugate, in which an anti-mesothelin antibody (anetumab) is attached to a tubulin inhibitor (ravtansine) [119].

As β1-integrins play a pivotal role in different mechanisms underlying progression of ovarian carcinoma, they are targeted in clinical trials in ovarian carcinoma. Volociximab, a chimeric monoclonal antibody that binds to and inhibits the functional activity of α5β1-integrins, was studied in a phase II trial as a monotherapy in patients with platinum-resistant advanced epithelial ovarian or primary peritoneal cancer. Although the agent alone did not provide sufficient clinical activity, it was well tolerated, prompting the development of improved strategies to target α5β1-integrins [120]. A phase II clinical trial (ClinicalTrials.gov identifier: NCT00635193) was conducted in patients with advanced ovarian cancer or those who relapsed after platinum/taxane therapy that tested volociximab in a combination with liposomal doxorubicin. Preliminary data from this trial suggest that the combination was well tolerated [121], while no data on the efficacy against the relapsed disease was reported.

Epidermal growth factor receptor (EGFR) is overexpressed in epithelial ovarian carcinoma and its high expression is associated with poor prognosis [122]. EGFR is important in progression of many cancer types [123]; thus, it became one of the major targets in cancer following the development of several targeting agents [124,125]. Therefore, many clinical trials addressed targeting EGFR using tyrosine kinase inhibitors or antibodies as monotherapy in ovarian cancer, although the results of these trials demonstrated no difference in survival [126]. While new EGFR-targeting agents, such as a monoclonal antibody matuzumab (EMD 72000), are still being investigated as monotherapy (ClinicalTrials.gov identifier: NCT00073541), combinations of EGFR-targeting therapy with the standard chemotherapy are being tested in other clinical trials. In one such combination trial, gefitinib (Iressa), a small-molecule inhibitor of EGFR, is being investigated in combination with topotecan, a topoisomerase inhibitor, in patients with relapsed ovarian, peritoneal, and fallopian tube cancers (ClinicalTrials.gov identifier: NCT00317772).

A small-molecule inhibitor of CXCR4, plerixafor (Mozobil), will be investigated in patients with advanced cancers, including ovarian (ClinicalTrials.gov identifier: NCT02179970). VEGFRs were studied as targets of angiogenesis in ovarian carcinoma, resulting in approval of a monoclonal antibody against VEGFA, bevacizumab (Avastin), by The Food and Drug Administration in 2014 [127]; however, these agents were not directed at targeting the lymphogenous spread.

In summary, although many targeted therapies directed at mechanisms described in this review are yet to show any advantage in the treatment of relapsed ovarian carcinoma when given as a monotherapy, optimism still remains that their combinations with other chemotherapeutic agents will benefit the patients at terminal stages of the disease. The successes behind targeted therapies for breast cancer gene (*BRCA*)-deficient ovarian carcinoma with the inhibitors of poly (ADP)-ribose polymerase [128] provide further confidence in the approach.

## 7. Conclusions

Many studies addressed mechanisms regulating the formation and development of intraperitoneal metastases. Foremost, the findings demonstrate the importance of the receptor–ligand interactions between metastatic EOC cells with other cells and molecules in the microenvironment, such as mesothelial cells, omental adipocytes, fibroblasts, ECM, and others, in the development of metastatic lesions. Disseminating EOC cells are endowed with expression of several types of receptors, including those for chemokines, tyrosine kinases, integrins, and glycoproteins. Expression of these receptors proved to be essential for successful colonization of the various tissues and organs. These findings pointed research efforts toward the development of targeted therapies against disseminating EOC cells. However, further studies into EOC metastasis-related mechanisms are crucial for the development of personalized therapies against this highly heterogeneous and deadly disease. Several studies highlighted the role of the microenvironment and stromal cells (both naïve and tumor-associated) in the intraperitoneal milieu, as well as the role of the molecular changes during aging that support peritoneal metastases. Thus, these causal aspects of the microenvironment should also be viewed as potential molecular targets for reduction of the metastatic spread and prevention of recurrences. Both hematogenous and lymphatic routes of dissemination are relatively less studied due to their perceived limited impact on the outcomes, as most EOC patients succumb to the intraperitoneal metastasis. Nonetheless, it is likely that their clinical relevance, however unfortunate for the patients, will increase as treatment of the intraperitoneal metastasis improves in the future. Therefore, understanding of the mechanisms regulating the distant metastasis is essential for ultimately blocking this deadly metastatic disease. Overall, a more comprehensive characterization of the mechanisms regulating metastatic ovarian carcinoma and therapy response is required for the development of new targeted therapies and improvement of currently used treatment regimens.

## Figures and Tables

**Table 1:** Prevalence of peritoneal metastasis in patients with epithelial ovarian carcinoma (EOC). USA—United States of America; UK—United Kingdom.

Patient Population, Institution	Number of Cases	Method of Study	Number of Cases with Peritoneal Metastasis (Percentage of Total)	Time of Assessment	Reference
USA, Roswell Park Institute	381*	Autopsy	316 (83%)	At autopsy	[27]
USA, University of Rochester Medical School	100	Autopsy	73 (73%)	At autopsy	[25]
USA, National Cancer Institute	73	Autopsy	39 (53%)	At autopsy	[26]
Switzerland, University of Basel	166 **	Autopsy	164 (99%)	At autopsy	[32]
UK, St. Bartholomew’s Hospital	67 ***	Computed tomography	59 (88%)	At relapse	[30]
Japan, The Jikei University School of Medicine	70 ****	Imaging, cytometry, CA125 level	49 (70%)	At relapse	[31]

* The number of cases with *epithelial* ovarian carcinoma only. ** The number of patients who were analyzed. *** The number of cases for which complete imaging data are available. **** The number of cases with recurrent ovarian cancer.

**Table 2:** Most frequently colonized lymph nodes identified in EOC patients by autopsy studies.

Study	Abdominal Lymph Nodes	Pelvic Lymph Node	Thoracic Lymph Node
[27]	58%	48%	28%
[25]	47%	17%	29%
[32]	74.1%	27.7	34.9
Average number of patients with indicated metastasis	60	31	31
